# Discovery and characterization of verinurad, a potent and specific inhibitor of URAT1 for the treatment of hyperuricemia and gout

**DOI:** 10.1038/s41598-017-00706-7

**Published:** 2017-04-06

**Authors:** Philip K. Tan, Sha Liu, Esmir Gunic, Jeffrey N. Miner

**Affiliations:** 1 Department of Biology, Ardea Biosciences, Inc. (A member of the AstraZeneca Group), San Diego, CA USA; 2 Department of Chemistry, Ardea Biosciences, Inc. (A member of the AstraZeneca Group), San Diego, CA USA

## Abstract

Gout is caused by elevated serum urate levels, which can be treated using inhibitors of the uric acid transporter, URAT1. Here, we characterize verinurad (RDEA3170), which is currently under evaluation for gout therapy. Verinurad specifically inhibits URAT1 with a potency of 25 nM. High affinity inhibition of uric acid transport requires URAT1 residues Cys-32, Ser-35, Phe-365 and Ile-481. Unlike other available uricosuric agents, the requirement for Cys-32 is unique to verinurad. Two of these residues, Ser-35 and Phe-365, are also important for urate transport kinetics. A URAT1 binding assay using radiolabeled verinurad revealed that distinct URAT1 inhibitors benzbromarone, sulfinpyrazone and probenecid all inhibit verinurad binding via a competitive mechanism. However, mutations made within the predicted transporter substrate channel differentially altered the potency for individual URAT1 inhibitors. Overall, our results suggest that URAT1 inhibitors bind to a common site in the core of the transporter and sterically hinder the transit of uric acid through the substrate channel, albeit with vastly different potencies and with differential interactions with specific URAT1 amino acids.

## Introduction

Gout is a metabolic disease caused by chronically elevated serum uric acid (sUA) levels (hyperuricemia), leading to deposition of urate in the joints and acute bouts of painful inflammatory arthritis^1, 2^. Urate homeostasis is balanced by urate production and elimination, and hominoids and certain monkeys have relatively high sUA levels due to the presence of multiple inactivating mutations in the uric acid degrading enzyme uricase and mutations in URAT1 that increase affinity for uric acid^3, 4^. Elimination of urate occurs primarily in the urine; however, in the kidneys approximately 90% of the urate filtered by the glomerulus is reabsorbed back into the bloodstream so that just 10% of the filtered urate is renally excreted^5, 6^. Compared to individuals with normal sUA levels, most patients with gout exhibit reduced fractional excretion of uric acid (FEUA), leading to hyperuricemia^7^. URAT1 is a transporter critical for renal reabsorption of urate. Inactivating mutations of URAT1 lead to high FEUA and hypouricemia (abnormally low sUA levels)^8^. In patients with gout, we previously hypothesized that reduced FEUA could be due to altered URAT1 transport kinetics that increase renal urate reabsorption^7^. However, the reduced FEUA in gout patients could also be due to reduced renal urate secretion^5, 6^.

Benzbromarone, sulfinpyrazone, probenecid and lesinurad (Figure S1) are among a class of gout therapeutics that lower sUA levels by inhibiting URAT1 and enhancing FEUA. However, the first three are not widely used due to various safety and availability issues^9^. Lesinurad was recently approved for the treatment of hyperuricemia associated with gout, in combination with a xanthine oxidase inhibitor^10–14^. In this report, we describe the molecular pharmacology of a novel, highly potent and specific URAT1 inhibitor, verinurad (also known as RDEA3170; Figure S1), that is currently under evaluation for the treatment of gout and asymptomatic hyperuricemia. Verinurad-mediated inhibition of URAT1 is highly dependent on human URAT1 Phe-365 and Ser-35, both of which are located in the substrate channel. The high affinity of verinurad allowed development of a novel URAT1 binding assay, and results from this assay showed that all the inhibitors bind to the same site within URAT1. Ser-35 and Phe-365 are also important in affinity for urate, suggesting that URAT1 inhibitors bind in the core of the transporter and sterically hinder the transit of uric acid through the substrate channel.

## Materials and Methods

### Reagents

Benzbromarone and sulfinpyrazone were obtained from Sigma-Aldrich. Verinurad, 2-((3-(4-cyanonaphthalen-1-yl)pyrindin-4-yl)thio)-2-methylpropanoic acid, and lesinurad, 2-((5-bromo-4-(4-cyclopropylnaphthalen-1-yl)-4H-1,2,4-triazol-3-yl)thio)acetic acid, were synthesized at Ardea Biosciences. These URAT1 inhibitors were diluted in DMSO at 20 or 100 mM concentrations. Water-soluble probenecid (Life Technologies) was prepared according to the manufacturer’s instructions. ^14^C-uric acid (50–60 mCi/mmol, 0.5 mCi/mL) was from American Radiolabeled Chemicals, Inc. ^3^H-verinurad was synthesized by Moravek Biochemicals with a specific activity of 21.3 Ci/mmol and a concentration of 1 mCi/ml, at a purity of 99%, with tritiated methyl groups. Supplementary Figure 1 shows the structure of these compounds.

### Constructs

Human URAT1 (GenBank BC053348.1, Homo sapiens *SLC22A12*), rat URAT1 (NCBI NM_001034943.1, Rattus norvegicus *Slc22a12*) and human OAT4 (NCBI NM_018484.2, Homo sapiens *SLC22A11*) genes were purchased from Origene Technologies, Inc. and subcloned into pCMV6/neo using *Not*I. Where indicated, controls transfections used pCMV6/neo. Mutants were produced by site-directed mutagenesis using the QuikChange Lightning Multi Site-Directed Mutagenesis Kit (Agilent Technologies). All mutants were confirmed by DNA sequencing. Mutagenic primer sequences are provided in ref. 15 and mutagenic primer sequences for previously unreported mutants shown in Supplementary Table 1. All constructs used in this study were untagged. Human OAT1 was purchased from Fisher Scientific (catalog # MHS1010–7508183 in pSPORT6, NCBI NM_153276.2, Homo sapiens *SLC22A6*).

### Cell culture and transfection

HEK-293T cells were maintained in DMEM/high glucose/L-glutamine/HEPES supplemented with 1 mM sodium pyruvate (Life Technologies) and 10% FBS (PAA Laboratories) at 37 °C in 5% CO_2_. To express URAT1, OAT4 or OAT1, plasmids were reverse transfected into HEK-293T cells. DNA (10 µg) was mixed with 30 µl of DreamFect Gold (Boca Scientific) in 2 ml OptiMem (Life Technologies). After 20 minutes, 18 ml of media containing 2 × 10^7^ cells in suspension was added and mixed, and 200 µl per well were plated onto white clear-bottomed poly-D-lysine coated 96-well plates (BD Biosciences). The cells were assayed the following day.

### URAT1 and OAT4 activity assays

URAT1 and OAT4 activity assays were performed in assay buffer consisting of 25 mM MES pH 5.5 (from a 1 M MES solution [Sigma] adjusted to pH 5.5 with sodium hydroxide), 125 mM sodium gluconate, 4.8 mM potassium gluconate, 1.2 mM monobasic potassium phosphate, 1.2 mM magnesium sulfate, 1.3 mM calcium gluconate and 5.6 mM glucose. URAT1 inhibitors were serially diluted into assay buffer and added to the cells for 5 minutes prior to addition of substrate. URAT1-expressing cells were incubated with 100 µM ^14^C-uric acid for 10 minutes and OAT4-expressing cells were incubated with 20 µM carboxyfluorescein (Life Technologies, Inc.) for 5 minutes. Cells were then washed three times in 25 mM MES pH 5.5/125 mM sodium gluconate, and solubilized in Ultima Gold (Perkin Elmer) prior to liquid scintillation counting. Each treatment was measured in triplicate.

### OAT1 activity assays

OAT1-expressing cells were assayed in a buffer containing 20 mM HEPES (from a 1 M solution at pH 7.3; USB Corporation), 130 mM sodium chloride, 4 mM potassium chloride, 1 mM magnesium sulfate, 1 mM calcium chloride, 1 mM sodium phosphate dibasic and 18 mM glucose. Cells without or with compounds were incubated with 20 µM carboxyfluorescein for 5 minutes, and then washed three times with 20 mM HEPES pH 7.3/130 mM sodium chloride. Cells were then solubilized in 0.1 N sodium hydroxide prior to fluorimetry measurements. Each treatment was measured in triplicate.

### Verinurad studies in humans

The study was conducted in accordance with Good Clinical Practice and the Declaration of Helsinki and with independent ethics committee (Welwyn Clinical Pharmacology Ethics Committee, Hatfield, UK) approval. Informed consent was obtained from all subjects before participating in the study.

Six healthy male volunteers with a mean age of 25 ± 7.6 years and body mass index of 23 ± 2.8 were orally administered a single 40 mg dose of verinurad while fasting. Serum samples were collected prior to dosing and at 6, 12 and 24 hours post verinurad administration to measure uric acid and creatinine concentrations. Urine samples were collected from 0 to 6 hours, 6 to 12 hours and 12 to 24 hours post dosing to measure the urinary excretion of uric acid and creatinine. FEUA was calculated using equation 1:

1 $${\rm{FEUA}}=([{\rm{urine}}\,{\rm{uric}}\,{\rm{acid}}]\times [{\rm{serum}}\,{\rm{creatinine}}])/([{\rm{serum}}\,{\rm{uric}}\,{\rm{acid}}]\times [{\rm{urine}}\,{\rm{creatinine}}])$$

To determine time-weighted average plasma verinurad concentrations (TWA), plasma verinurad levels were first measured from serial plasma collections. The weighted average plasma verinurad was then calculated for each timed-urine collection period (UCP, 0 to 6 hours, 6 to 12 hours and 12 to 24 hours) using equation 2:

2 $$\begin{array}{rcl}{\rm{TWA}} & = & [({{\rm{c}}}_{{\rm{1}}}+{{\rm{c}}}_{{\rm{2}}})/2\times ({{\rm{t}}}_{{\rm{2}}}-{{\rm{t}}}_{{\rm{1}}})]+[({{\rm{c}}}_{{\rm{2}}}+{{\rm{c}}}_{{\rm{3}}})/2\times ({{\rm{t}}}_{{\rm{3}}}-{{\rm{t}}}_{{\rm{2}}})]\\  &  & +\ldots +[({{\rm{c}}}_{{\rm{n}}-{\rm{1}}}+{{\rm{c}}}_{{\rm{n}}})/2\times ({{\rm{t}}}_{{\rm{n}}}-{{\rm{t}}}_{{\rm{n}}-{\rm{1}}})]/\mathrm{UCP}\end{array}$$

where c_1_ is the plasma concentration of verinurad at the first time interval, t_1_ is the first time interval, c_2_ is the plasma concentration of verinurad at the second time interval, t_2_ is the second time interval, etc.

### Binding assay

Membranes were prepared from cells transfected on the previous day with URAT1 expression constructs. Cells (5–10 × 10^8^) were harvested in 1 ml ice-cold binding buffer (25 mM HEPES pH 7.3, 125 mM sodium gluconate) containing complete, EDTA-free protease inhibitor cocktail (Sigma). Cells were lysed with 100 strokes in a 2 ml dounce homogenizer using a tight-fitting “B” stem. Lysates were transferred to 1.5 mL Eppendorf tubes and centrifuged at 250 rcf for 5 minutes at 2 °C, and supernatants were transferred to fresh tubes and centrifuged again at 18,000 rcf for 20 minutes at 2 °C. The pellets (membrane fractions) were resuspended in 1 ml of ice-cold binding buffer containing protease inhibitors and frozen at −80 °C. Protein was measured using the Bio-Rad protein assay dye reagent concentrate.

To initiate binding, membranes (2.5 µg total protein) were incubated with ^3^H-verinurad without or with inhibitors for 30 minutes at room temperature. Samples were then rapidly filtered through 25 mm glass fiber/1.2 µM PES 96-well filter plates (Corning FiltrEX 3510), and washed once with 200 µl ice-cold binding buffer. The plates were then subjected to scintillation counting. All samples were assayed in triplicate. The inhibition constant was calculated from the equation for competitive inhibition (equation 3):

3 $${K}_{{\rm{i}}}={K}_{{\rm{m}}}[{\rm{I}}]/({K}_{{\rm{m}}(\mathrm{app})}-{K}_{{\rm{m}}})$$

### Non-linear regression and statistical analyses

GraphPad Prism software was used for non-linear regression and statistical analyses. The potencies of URAT1 inhibitors (half maximal inhibitory constant [IC_50_] values) were obtained from the equation “log (inhibitor) versus response - variable slope (four parameters)”, while the *in vivo* potency of verinurad on the increase in FEUA was obtained from the equation “log (agonist) versus response - variable slope (four parameters)”. For kinetic measurements (*K*_m_ and *B*_max_ values), the “one site – specific binding” equation was used, after subtracting transport or binding measurements from control transfected cells. Statistical significance was assessed using Student’s unpaired *t*-tests.

## Results

### Verinurad, a highly potent and specific URAT1 inhibitor

**Figure 1:**
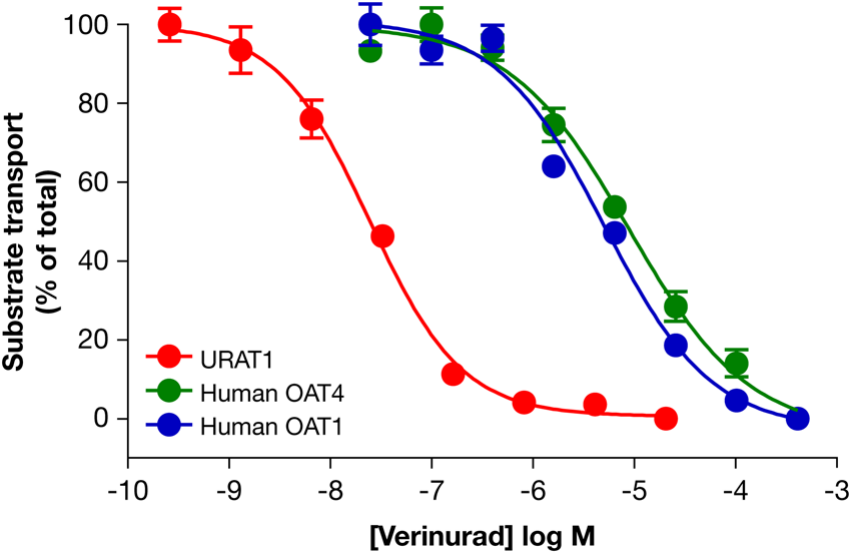
Verinurad is highly potent and specific for human URAT1. Dose-responses for verinurad against the transport activity of human URAT1 (red), human OAT4 (green) and human OAT1 (blue). Cells expressing URAT1 were incubated with ^14^C-uric acid, and cells expressing OAT4 or OAT1 were incubated with carboxyfluorescein, in the presence of different amounts of verinurad. Data are from one representative experiment presented as the mean ± SEM of samples measured in triplicate.

**Figure 2:**
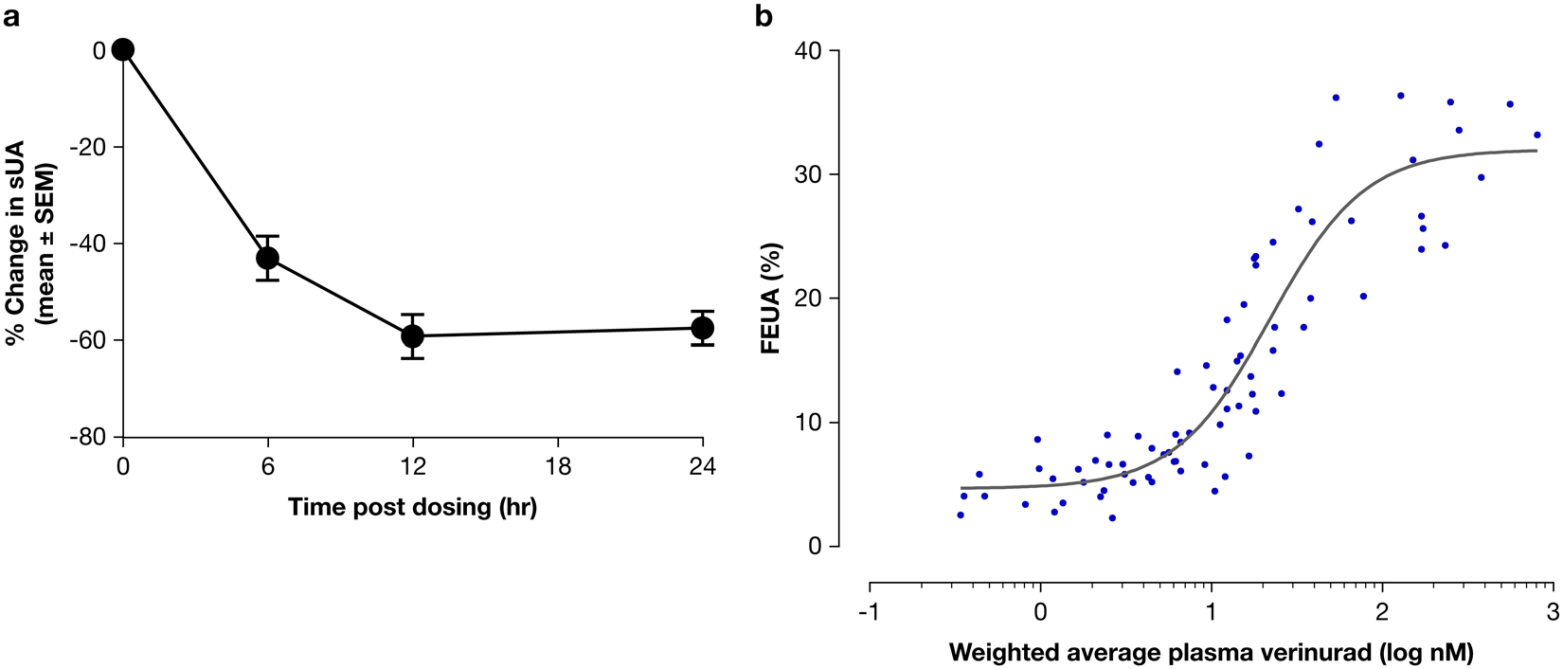
Verinurad lowers serum uric acid (sUA) by increasing fractional excretion of uric acid (FEUA) in humans. (**a**) A single 40 mg dose of verinurad lowered the baseline sUA levels by up to 60% for a sustained time period. (**b**) Verinurad increased the FEUA in a dose-dependent manner, with a half-maximal effective plasma concentration 22 nM. Experiments were performed in healthy human volunteers.

Verinurad inhibited the transport activity of human URAT1 in a dose-dependent manner, at high potency with an IC_50_ of 25 nM (Fig. 1, red curve and Table 1). Verinurad inhibited the related URAT1 homologs OAT4 (Fig. 1, green curve) and OAT1 (Fig. 1, blue curve) with approximately 200-fold lower affinity compared to URAT1 with IC_50_ values of 5.9 µM and 4.6 µM, respectively (Table 1). Therefore, verinurad is highly specific for URAT1. Consistent with the *in vitro* inhibition of URAT1, treatment of healthy human volunteers with a single 40 mg dose of verinurad reduced the sUA by up to 60% (Fig. 2a) and increased the FEUA in a dose-dependent manner with a half-maximal effective concentration in plasma of 22 nM (Fig. 2b).

**Table 1:** Potency of verinurad against human URAT1, human OAT4, human OAT1 and rat URAT1. Values (mean ± SEM) are from at least three dose-response experiments as shown in Fig. 1. Asterisks indicate significant differences in the mean value from human URAT1. **p < 0.01, ***p < 0.001, ****p < 0.0001.

	Human URAT1	Human OAT4	Human OAT1	Rat URAT1
% Homology to human URAT1	100	51	46	74
Verinurad affinity (IC_50_, µM)	0.025 ± 0.0036	5.9 ± 1.4***	4.6 ± 1.4**	41 ± 2.7****

### Verinurad has a high potency for human URAT1, and residues 35, 365 and 481 all contribute to verinurad affinity

**Figure 3:**
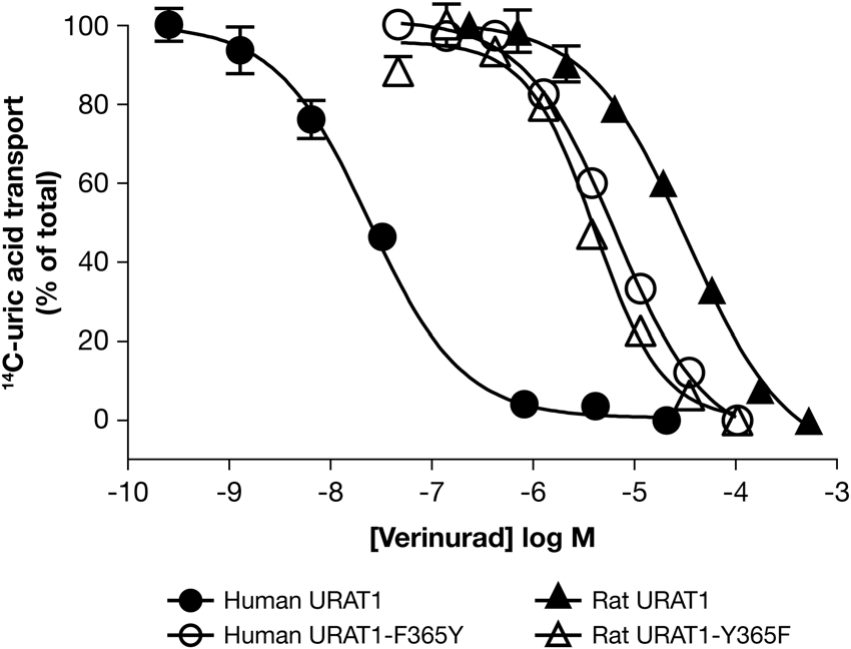
Phe-365 of human URAT1 is important for affinity for verinurad. Dose-response curves of verinurad against human URAT1 (solid circles), human URAT1-F365Y (open circles), rat URAT1 (solid triangles) and rat URAT1-Y365F (open triangles). The experiment was performed as described in Fig. 1. The potency of verinurad for each construct can be found in Table 2. Data are from one representative experiment presented as the mean ± SEM of samples measured in triplicate.

Human URAT1 has a higher affinity for URAT1 inhibitors compared to rat URAT1, and this difference is mediated by human URAT1 residues Ser-35, Phe-365 and Ile-481, located in transmembrane (TM) segments 1, 7 and 11, respectively, which are Asn-35, Tyr-365 and Met-481 in rat URAT1, respectively^15^. Since verinurad also has a higher potency for human URAT1, we analyzed point mutant chimeras at these positions to determine if these residues are also important for the affinity for verinurad (Table 2). Point mutations swapping human and rat residues at positions 35, 365 and 481 all produced phenotypes, with chimeras at residue 365 producing the strongest phenotypes (Fig. 3). Human URAT1 (Fig. 3, filled circles; Table 1, IC_50_ = 0.025 µM) had a 1,640-fold higher affinity for verinurad compared to rat URAT1 (Fig. 3, filled triangles; Table 1, IC_50_ = 41 µM). Human URAT1 carrying a chimeric point mutation at position 365, in which human Phe-365 is replaced by rat Tyr-365 (human URAT1-F365Y or h-F365Y) had an IC_50_ of 4.0 µM (Fig. 3, open circles), a significant 160-fold lower affinity relative to human URAT1 (Table 2). Meanwhile, rat URAT1 carrying the opposite point mutation (rat URAT1-Y365F or r-Y365F), had an IC_50_ of 2.9 µM (Fig. 3, open triangles), a significant 14-fold *higher* affinity compared to rat URAT1 (Table 2).

**Table 2:** Potency of verinurad against human (h) URAT1 and rat (r) URAT1, and chimeric point mutants in TM1, TM7 and TM11.

	Human URAT1	Verinurad affinity (IC_50_, µM)	Rat URAT1	Verinurad affinity (IC_50_, µM)
Wild type	hURAT1	0.025 ± 0.0036	rURAT1	41 ± 2.7
TM1 point mutants	h-V18L	0.026 ± 0.015	r-L18V	40 ± 6.1
	h-T21A	0.026 ± 0.012	r-A21T	36 ± 5.2
	h-M22V	0.029 ± 0.013	r-V22M	43 ± 2.5
	h-M25V	0.029 ± 0.010	r-V25M	18 ± 3.2**
	h-V26T	0.014 ± 0.0015	r-T26V	39 ± 5.5
	h-S27P	0.039 ± 0.0090*	r-P27S	27 ± 3.4**
	h-M29L	0.011 ± 0.0029**	r-L29M	29 ± 3.3*
	h-L31V	0.031 ± 0.010	r-V31L	29 ± 3.2*
	h-C32T	0.095 ± 0.026****	r-T32C	29 ± 3.1*
	h-S35N	0.36 ± 0.15***	r-N35S	10 ± 1.5****
TM7 point mutant	h-F365Y	4.0 ± 1.7***	r-Y365F	2.9 ± 1.3****
TM11 point mutant	h-I481M	0.053 ± 0.010***	r-M481I	11 ± 3.1***

Values (mean ± SEM) are from at least three dose-response experiments as shown in Fig. 3. Asterisks indicate significant differences in mean values from the corresponding wild type transporter. *p < 0.05, **p < 0.01, ***p < 0.001, ****p < 0.0001.

Chimeras at positions 35 and 481 also produced significant phenotypes (Table 2). At amino acid 35, h-S35N had a 14-fold lower affinity for verinurad relative to human URAT1, whereas r-N35S had a 4-fold *higher* affinity for verinurad relative to rat URAT1, demonstrating the importance of Ser-35 in human URAT1 for the affinity for verinurad. At residue 481, h-I481M showed a 2-fold reduction in verinurad affinity compared to human URAT1, while r-M481I showed a 4-fold *increase* in verinurad affinity compared to rat URAT1. Therefore, along with Phe 365, human URAT1 residues Ser-35 and Ile-481 are also involved in the high affinity interaction with verinurad.

**Figure 4:**
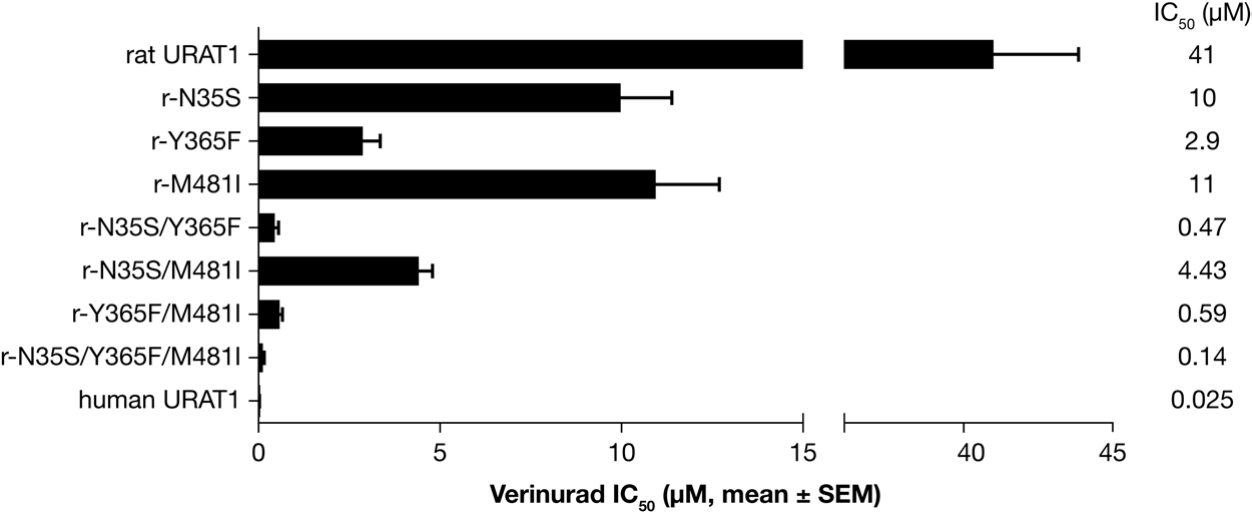
Human URAT1 residues 35, 365 and 481 cooperate to increase affinity for verinurad. Potency of verinurad against rat URAT1, individual or combination chimeric point mutants with human URAT1 residues at positions 35, 365 and 481, and human URAT1. Dose-response experiments were performed as in Fig. 3, and results are the mean ± SEM from at least three experiments.

To test potential cooperativity among these point mutations, we analyzed double and triple point mutant chimeras that have human URAT1 amino acids Ser-35, Phe-365 and Ile-481 in the context of rat URAT1 (Fig. 4). Rat URAT1 carrying double point mutation combinations of the human URAT1 residues all had an increased affinity relative to the corresponding single point mutants, while the triple point mutant r-N35S/Y365F/M481I had the highest affinity of all the rat constructs to verinurad. This construct had nearly a 300-fold higher affinity for verinurad compared to rat URAT1, and just a 6-fold lower affinity compared to human URAT1. Therefore, these amino acids functionally cooperate to increase the affinity for verinurad.

In addition to residue 35, TM1 has nine other non-conserved residues between human and rat URAT1. Therefore, point mutant chimeras of these residues were also analyzed for verinurad affinity (Table 2). These results indicate that human URAT1 Met-25 and Ser-27 are also involved in mediating verinurad affinity. r-V25M and r-S27P had a significant *increase* in affinity of 2.3- and 1.5-fold, respectively, while h-S27P had a significant 1.6-fold decrease in affinity. In addition, h-C32T had a significant 4-fold reduced affinity for verinurad relative to human URAT1, while r-T32C had a slight but still significant 1.4-fold *increase* in affinity for verinurad relative to rat URAT1.

### Human URAT1 Ser-35 and Phe-365 also increase affinity for urate

**Figure 5:**
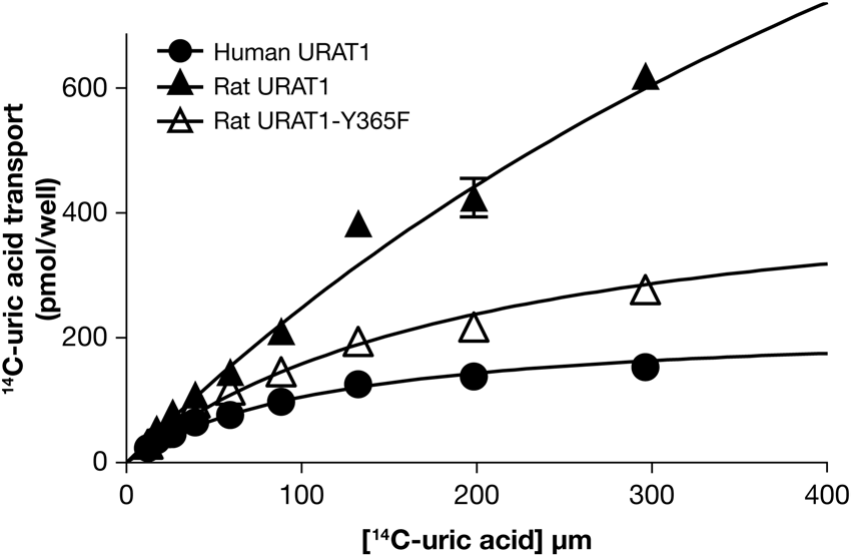
Human URAT1 Ser-35 and Phe-365 cooperate to enhance urate affinity. Transport kinetics of human URAT1 (filled circles), rat URAT1 (filled triangles) and the double point mutant chimera rat URAT1 carrying human URAT1 residues at 35 and 365 (r-N35S/F365Y, open triangles). Data are from one representative experiment presented as the mean ± SEM of samples measured in triplicate. The half maximal transport velocity (*K*_m_) for each construct is presented in Table 3.

Earlier studies showed that compared to rat URAT1, human URAT1 has a higher affinity for urate, and that human URAT1 Ser-35 and Phe-365 play a role in urate affinity^4, 15^. In this report, we extended these studies and examined the urate transport kinetics of the double point mutant rat URAT1-N35S/Y365F, along with the corresponding single point mutants and the wild type human and rat transporters (Table 3 and Fig. 5). Human URAT1 (Fig. 5, filled circles) had a significant 7-fold higher affinity for urate relative to rat URAT1 (Fig. 5, filled triangles; *K*_m_ = 122 and 857 µM, respectively). The single point mutant rat URAT1-N35S had no phenotype, while the single point mutant rat URAT1-Y365F had a significant 1.5-fold *increase* in urate affinity over rat URAT1 (Table 3). Meanwhile, the double point rat URAT1-N35S/Y365F (Fig. 5, open triangles) had a stronger phenotype relative to the corresponding single point mutants, with nearly 4-fold *increase* in affinity relative to rat URAT1 (Table 3). Therefore, in addition to contributing to verinurad affinity, human URAT1 Ser-35 and Phe-365 functionally cooperate to increase the affinity for urate.

**Table 3:** Summary of uric acid transport affinities of human URAT1, rat URAT1 and rat URAT1 point mutants at residues 35 and 365.

Construct	*K*_m_ (µM)
Human URAT1	122 ± 3
Rat URAT1	857 ± 6****
Rat URAT1-N35S	697 ± 93
Rat URAT1-Y365F	560 ± 28^††^
Rat URAT1-N35S/Y365F	222 ± 16^††††^

Affinities in *K*_m_ were obtained from saturation transport curves as in Fig. 5, and are the mean ± SEM of at least three experiments. Data for human URAT1, rat URAT1 and rat URAT1-Y365F were previously reported in Tan *et al*., 2016. Statistical analyses are based on unpaired, two-tailed tests. **p < 0.01, ****p < 0.0001, compared to human URAT1. ^††^ *p* < 0.01, ^††††^ *p* < 0.0001 compared to rat URAT1.

### Each URAT1 inhibitor requires common and distinct residues for high affinity interaction with URAT1

**Figure 6:**
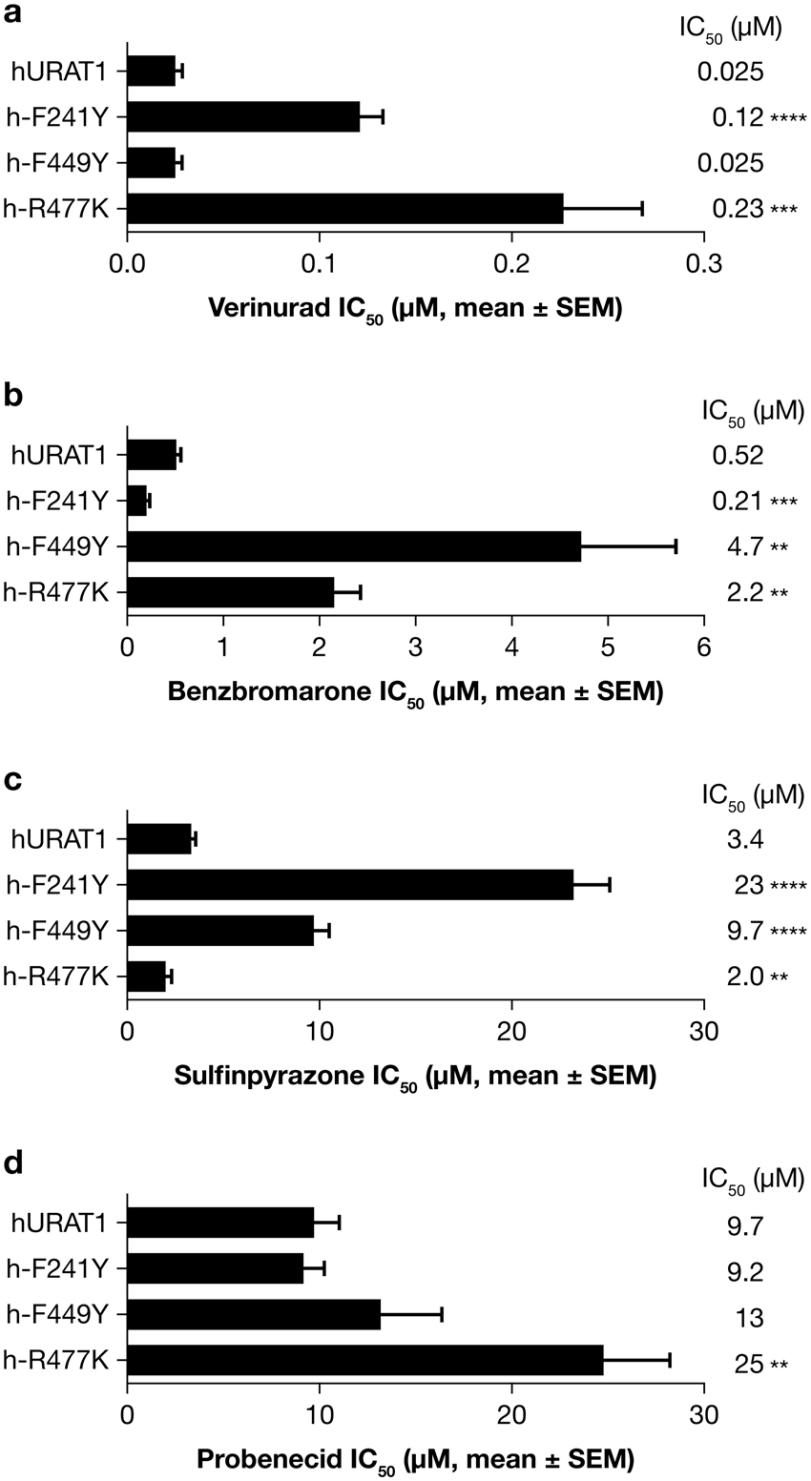
Each URAT1 inhibitor has a distinct profile of potencies for URAT1 mutants. Potencies of verinurad (**a**), benzbromarone (**b**), sulfinpyrazone (**c**) and probenecid (**d**) against human URAT1 (hURAT1) and hURAT1 with “binding site” point mutants F241Y, F449Y and R477K. Dose-response experiments were performed as in Fig. 3, and results are the mean ± SEM from at least three experiments.

When the human URAT1 residues Ser-35, Phe-365 and Ile-481 were visualized in a computer model of human OAT1^16^, the sidechains were in close proximity within the transporter channel, indicating that they form a binding site for inhibitors and urate^15^. To further characterize the binding site, we analyzed mutations in other residues that are also predicted to be within the transporter channel, based on prior studies with OAT1^16, 17^. In particular, we focused on Phe-241 in TM5, Phe-449 in TM10 and Arg-477 in TM11, which are conserved in human and rat URAT1. We made conservative point mutations for each of these residues in human URAT1 and analyzed their affinities for verinurad as well as for benzbromarone, sulfinpyrazone and probenecid (Fig. 6). With verinurad (Fig. 6a), h-F241Y had a 5-fold reduced affinity, h-F449Y had the same affinity, and h-R477K had a 9-fold reduced affinity compared to human URAT1 (hURAT1). In contrast, with benzbromarone (Fig. 6b), h-F241Y had a 2-fold *increased* affinity, h-F449Y had a 9-fold reduced affinity and h-R477K had a 4-fold reduced affinity compared to hURAT1. Therefore, the profile of responses between the two inhibitors differs for these amino acid substitutions. For verinurad, h-R477K has the largest difference in affinity, while h-F449Y has no phenotype. For benzbromarone, h-F449Y has the largest difference in affinity. Sulfinpyrazone (Fig. 6c) also has a distinct response profile, with h-F241Y showing the largest difference in affinity. Compared to hURAT1, h-F241Y had a 7-fold reduced affinity, h-F449Y had a 3-fold reduced affinity and h-R477K had a 2-fold *increased* affinity for sulfinpyrazone. With probenecid (Fig. 6d), only h-R477K had a significant 3-fold reduced affinity compared to hURAT1. The distinct response profiles of each inhibitor with this panel of mutants illustrate the complexity of the interactions with URAT1.

### Inhibitors all bind to the same site on human URAT1

**Figure 7:**
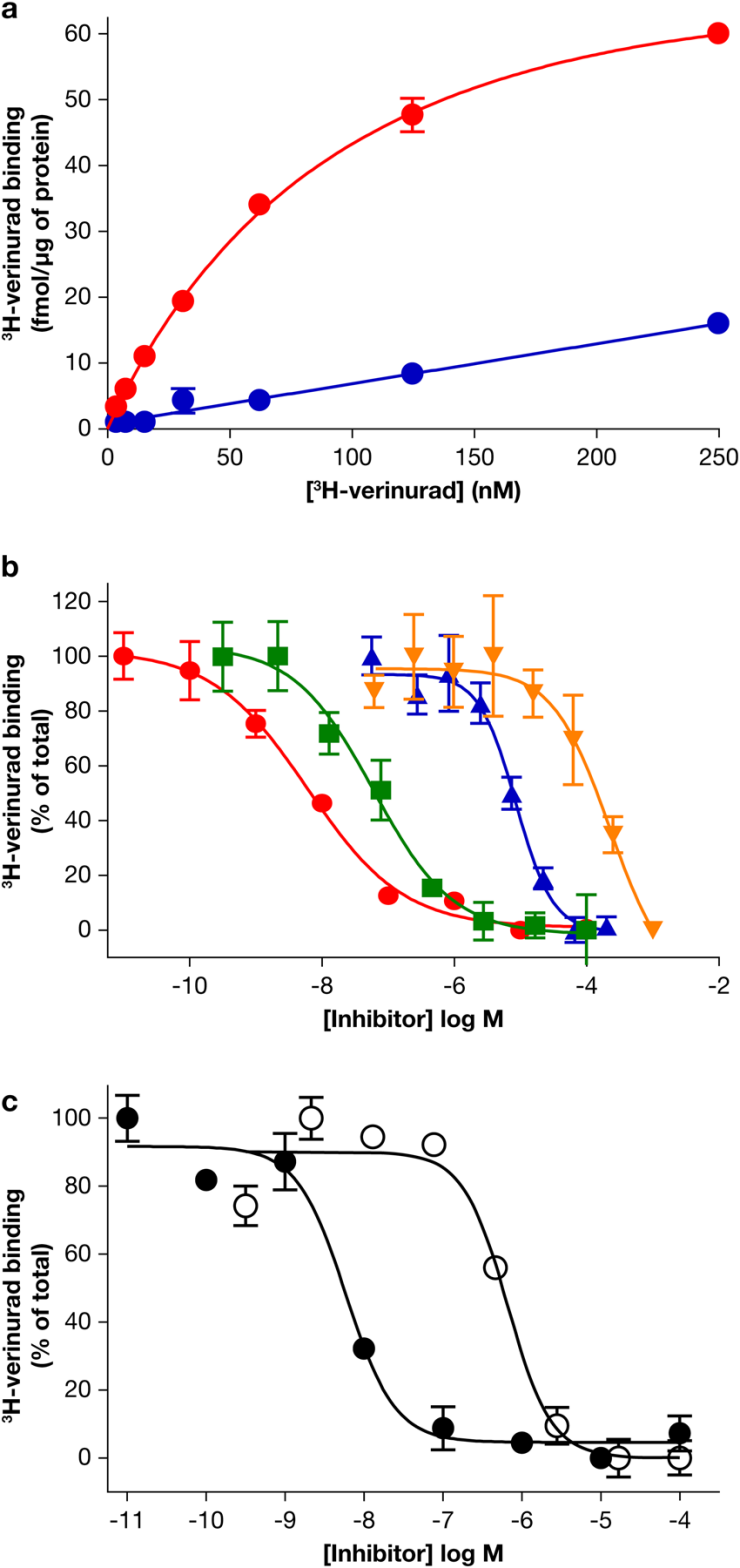
A novel URAT1 binding assay replicates the results of inhibition of URAT1 activity. (**a**) ^3^H-verinurad bound specifically to human URAT1. ^3^H-verinurad bound to membranes from cells transfected with human URAT1 in a specific and saturable manner (red) but not to membranes from cells transfected with empty vector (blue). (**b**) ^3^H-verinurad binding to human URAT1 was blocked by URAT1 inhibitors in a dose-dependent manner. 10 nM of ^3^H-verinurad was incubated with human URAT1 membranes and different concentrations of unlabeled verinurad (red), benzbromarone (green), sulfinpyrazone (blue) and probenecid (orange). (**c**) ^3^H-verinurad bound to membranes from cells transfected with human URAT1-F449Y, which was blocked in a dose-dependent manner by unlabeled verinurad (filled circles) and by benzbromarone (open circles). Compared to human URAT1, h-F449Y has the same affinity for verinurad but an 11-fold lower affinity for benzbromarone, mimicking the results for the activity assay (Fig. 6).

**Figure 8:**
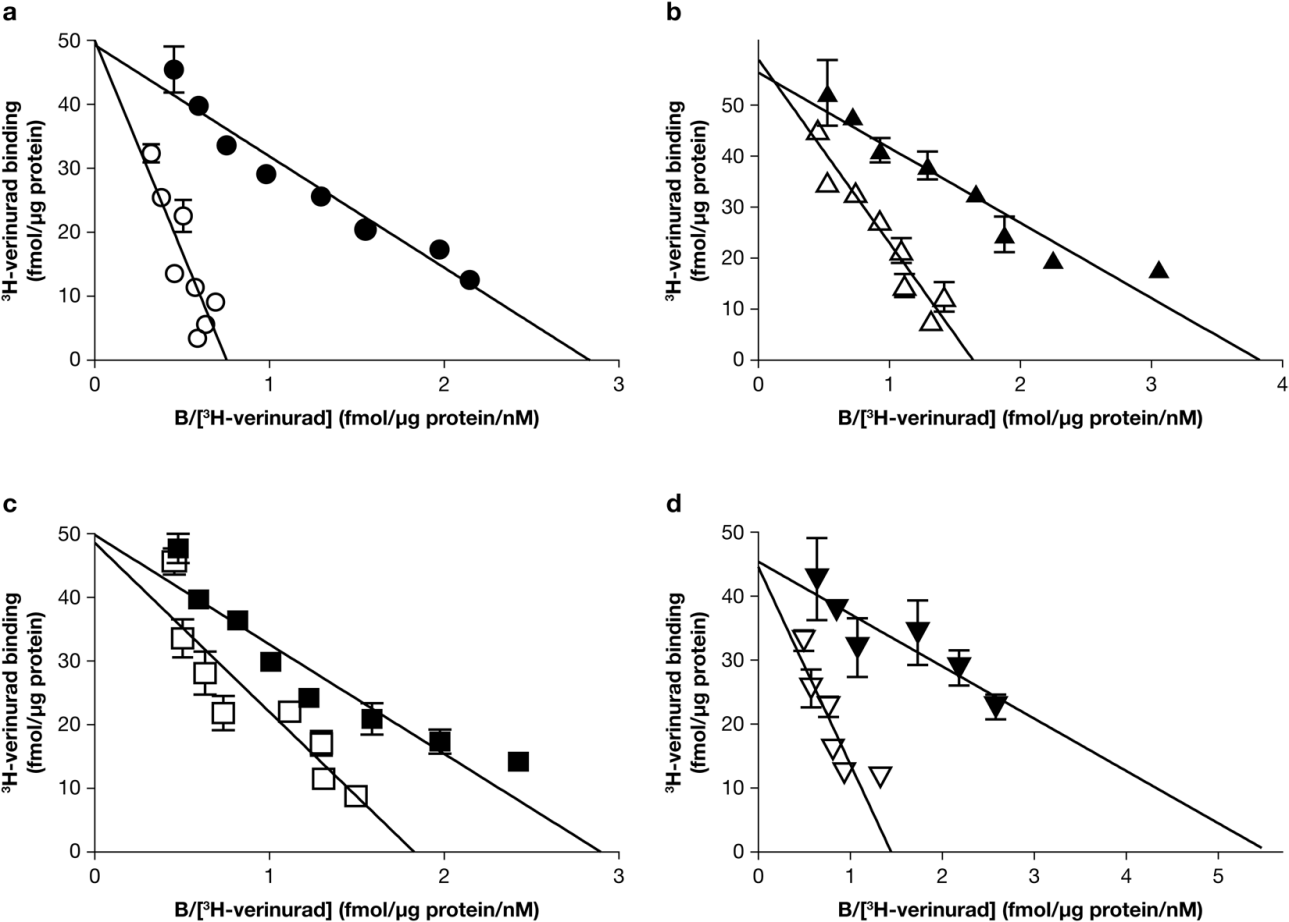
Inhibitors bind to the same binding site on URAT1. Binding of ^3^H-verinurad to human URAT1 in the absence of inhibitors (filled symbols) or in the presence of 20 nM unlabeled verinurad (**a**) (open circles), 50 nM benzbromarone (**b**) (open upward triangles), 25 µM sulfinpyrazone (**c**) (open squares) or 200 µM probenecid (**d**) (open downward triangles). Membranes containing human URAT1 were incubated with different concentrations of ^3^H-verinurad without or with inhibitors. Specifically bound ^3^H-verinurad is shown by Eadie-Hofstee linearization. B = bound verinurad.

Likely due to the high affinity of verinurad for human URAT1, we were able to develop a novel URAT1 binding assay using radiolabeled verinurad (Fig. 7; ref. 15). ^3^H-verinurad bound specifically to human URAT1 (Fig. 7a, red curve) with an affinity (*K*_m_) of 21 ± 1.9 nM and did not specifically bind to control membranes lacking human URAT1 (Fig. 7a, blue curve). The binding of ^3^H-verinurad to human URAT1 was inhibited in a dose-dependent manner by unlabeled verinurad (Fig. 7b, red curve, IC_50_ = 7.8 nM), benzbromarone (Fig. 7b, green curve, IC_50_ = 60 nM), sulfinpyrazone (Fig. 7b, blue curve, IC_50_ = 8.6 µM) and probenecid (Fig. 7d, orange curve, IC_50_ = 223 µM). Compared to the URAT1 transport assay (Fig. 6), the potencies (IC_50_ values) of benzbromarone and probenecid differ in the binding assay, which could be due to the different buffers used for each assay (see Materials and Methods). Consistent with the functional URAT1 activity assay (Fig. 6a), binding of ^3^H-verinurad to h-F449Y was inhibited by unlabeled verinurad with a potency (IC_50_) of 5.9 nM (Fig. 7c, filled circles), similar to the potency of verinurad against wild type human URAT1 (Fig. 7b, red curve). Meanwhile, binding of ^3^H-verinurad to h-F449Y was inhibited by benzbromarone with a potency of 0.65 µM (Fig. 7c, open circles), which is an 11-fold reduced potency compared to the inhibition of verinurad binding to wild type human URAT1 (Fig. 7b, green curve). This difference in affinity for benzbromarone between human URAT1 and h-F449Y resembled the differences in affinity for benzbromarone between these transporters in the urate activity assay (Fig. 6b). Overall, the results for the inhibitors using this URAT1 binding assay replicate the findings for the inhibitors in the URAT1 activity assay, indicating that functional inhibition of URAT1 activity by verinurad is due to direct binding. Supporting this statement, when the kinetics of ^3^H-verinurad binding to URAT1 were measured in the presence of inhibitors, a competitive mode of inhibition was detected (Fig. 8). Compared with untreated URAT1 samples (Fig. 8a, filled circles, *K*_m_ = 21 nM, *B*_max_ = 53 fmol/µg protein), samples treated 20 nM unlabeled verinurad showed an increase in the apparent affinity while the apparent total binding was unchanged (Fig. 8a, open circles, *K*_m(app)_ = 82 nM, *B*_max(app)_ = 59 fmol/µg protein), indicative of the expected competitive inhibition of the unlabeled identical compound. Similarly, benzbromarone (Fig. 8b, open upward triangles), sulfinpyrazone (Fig. 8c, open squares) and probenecid (Fig. 8d, open downward triangles) all showed a competitive mode of inhibition, with *K*_m(app)_ = 37, 36 and 43 nM, and *B*_max(app)_ = 60, 55 and 53 fmol/µg protein, respectively. Therefore, all of these inhibitors interact competitively at the same binding site on URAT1. The inhibition constant (*K*_i_) for unlabeled verinurad, benzbromarone, sulfinpyrazone and probenecid was 6.9 nM, 45 nM, 52 µM and 50 µM, respectively.

## Discussion

Verinurad is a novel, highly potent, and specific URAT1 inhibitor currently in phase 2 trials for the treatment of gout and asymptomatic hyperuricemia. This compound is one of the most potent URAT1 inhibitors yet identified and is highly specific for URAT1^18–20^, with greater than 100-fold potency for URAT1 compared to other transporters. In patients, verinurad is also extremely potent: a single 40 mg dose lowers serum uric acid by 60% and maintains sUA below pretreatment levels for 24 hours. In fact, verinurad significantly lowers sUA levels at doses as low as 5 mg^21^. The FEUA in these patients is rapidly increased after dosing, suggesting that the mechanism of action is via direct inhibition of tubular reabsorption of uric acid.

The high potency of verinurad provided the opportunity to more completely define the molecular interactions of inhibitors with URAT1. To aid in this endeavor, we developed the first URAT1 binding assay using radiolabeled verinurad^15^, which established that verinurad and the other inhibitors benzbromarone, sulfinpyrazone and probenecid all bind directly to the same site on URAT1 (competitive inhibition of verinurad binding). Moreover, the high specificity of verinurad, with more than a 1000-fold higher affinity for human URAT1 over rat URAT1, also enabled further characterization of the amino acids that are important for the interaction of inhibitors with human URAT1. Analysis of human-rat URAT1 chimeras indicated a clear role of Phe-365 in human URAT1, which is Tyr-365 in rat URAT1, for the affinity for verinurad. The dramatic 14-fold increase in affinity of the rat URAT1 carrying the human Phe-365 (Fig. 3), a gain-of-function phenotype, suggests that Phe-365 forms a direct and strong interaction with verinurad, possibly through hydrophobic π-π interactions^22^ between the phenyl-ring of Phe-365 and an aromatic group of verinurad. Consistent with this, Phe-365 is required for binding of ^3^H-verinurad to URAT1^15^. These interactions may be disrupted by the hydroxyl group of the rat URAT1 Tyr-365 benzyl-ring, explaining the lower affinity of rat URAT1. Phe-365 is also important for the affinity for other URAT1 inhibitors^15^, suggesting that Phe-365 directly binds to the aromatic moieties of the other inhibitors as well (Figure S1).

Verinurad has a higher potency against URAT1 compared to previously analyzed inhibitors^15^. In addition to Phe-365, analysis of human-rat URAT1 point mutants identified that human URAT1 Met-25, Ser-27, Cys-32, Ser-35 and Ile-481 also play a role in the interaction with verinurad. Cys-32 is the only residue that was not previously identified with other inhibitors. This residue along with Met-25, Ser-27 and Ser-35 all occur in TM1, implicating an important role for this TM in verinurad affinity. In a computer model of the URAT1 homolog OAT1^16^, the sidechains Ser-35, Phe-365 and Ile-481 are all in close proximity and project within the transporter channel^15^, suggesting that these residues form an inhibitor binding site, which is consistent with the strong phenotype observed when mutations in these residues are combined (Fig. 4). Within the structure of a TM α-helix, Cys-32 should also be in close proximity to Ser-35 and this inhibitor binding site. Consistent with the findings that these residues occur within the transporter channel, we found that Ser-35 and Phe-365 of human URAT1 are also important for the high affinity transport kinetics of the substrate urate. Therefore, it is likely that inhibitors block transport through steric hindrance of the passage of substrate through the transporter channel.

To further define the molecular interactions of URAT1 inhibitors, we analyzed point mutants of human URAT1 in other residues that are predicted to occur within the transporter channel and mediate substrate transport (Phe-241 in TM5 and Phe-449 in TM10, corresponding to Tyr-230 and Phe-438 in OAT1^16^; and Arg-477 in TM11 corresponding to Arg-466 in OAT1^17^, also see Supplementary Figure 2 for alignment of human URAT1 with OAT1 and other homologs). Unique responses were obtained for each URAT1 inhibitor (Fig. 6). For example, compared to wild type human URAT1, h-F449Y (human URAT1 with Phe-449 converted to Tyr) had the same affinity for verinurad but a lower affinity for benzbromarone and sulfinpyrazone. In contrast, h-F241Y had a lower affinity for verinurad and sulfinpyrazone and a *higher* affinity for benzbromarone. Meanwhile, h-R477K had a lower affinity for verinurad, benzbromarone and probenecid, and a *higher* affinity for sulfinpyrazone. These findings illustrate the complexity of inhibitor binding to URAT1, revealing unique interactions due to differences in the molecular shape and electron geometry among the compounds (Figure S1). Thus, each inhibitor interacts at a fundamentally common binding site but utilizes distinct residues from different TM domains that likely line the URAT1 transporter channel. Similarly, within the solute carrier transporter family, recent findings on the crystal structures of the glucose transporters show that residues from multiple TM segments interact with substrates^23–25^, and computer simulations of OAT1 transport showed conformational changes in many TM domains including TM1 and TM7^26^ further supporting our molecular analysis of URAT1.

We recently found that Tyr-365 was altered to Phe-365 in URAT1 during the evolution of simians (humans, apes and certain monkeys) resulting in enhanced affinity for urate transport^4^. This event was a possible adaptive mutation in human evolution to raise and more tightly control sUA levels. Interestingly, tyrosine residues occur in non-simian URAT1 orthologs as well as in the corresponding residues of most of the human URAT1 homologs such as OAT1, OAT3 (*SLC22A8*) and OAT4^15^ (see Supplementary Figure 2 for alignment of human URAT1 with the OAT homologs). Phe-365 is therefore a distinguishing feature of human URAT1 that is also required for high affinity urate and URAT1 inhibitor affinity. It is likely that the new highly potent acidic sulfonamides^19^ also require URAT1 Phe-365 for high affinity interaction, similar to verinurad.

Verinurad is a new highly potent and specific URAT1 inhibitor that reduces sUA levels and is currently under evaluation for the treatment of gout and asymptomatic hyperuricemia. The potency and selectivity of verinurad also provide a novel research tool for the investigation of the molecular mechanism of inhibition of URAT1, an important transporter that controls sUA levels in humans.

## Electronic supplementary material

Supplementary material
