# Supplementary material for: Discovery and characterization of verinurad, a potent and specific inhibitor of URAT1 for the treatment of hyperuricemia and gout
Source: Sci Rep. 2017 Apr 6;7:665. doi: 10.1038/s41598-017-00706-7 (PMC5429603; doi:10.1038/s41598-017-00706-7)
Supplement: Supplementary file 1 — Supplementary material [file 41598_2017_706_MOESM1_ESM.pdf]

Discovery and characterization of verinurad, a potent and specific inhibitor of URAT1 for the treatment of hyperuricemia and gout

Philip K. Tan[1,*], Sha Liu[1], Esmir Gunic[2] & Jeffrey N. Miner[1]

Fig S1. Structure of URAT1 inhibitors used in this study.

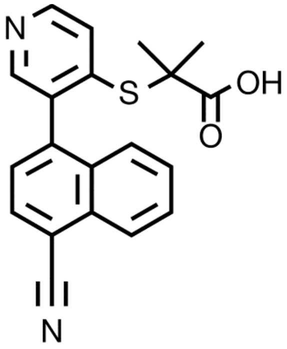

Verinurad

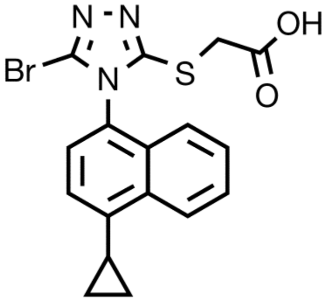

Lesinurad

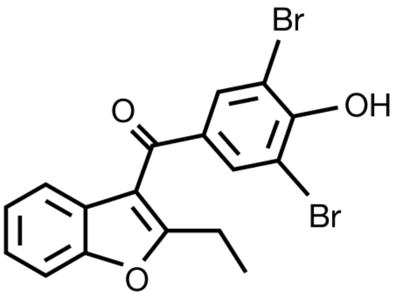

Benzbromarone

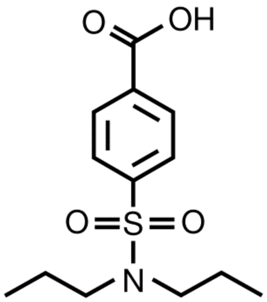

Probenecid

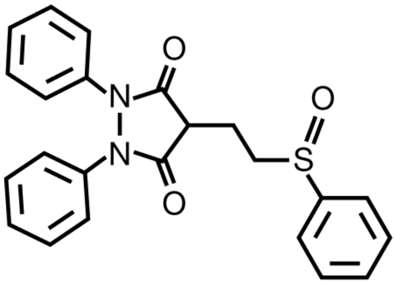

Sulfinpyrazone

Supplementary Table 1: **Sequences of mutagenic primers used for the production of h-F241Y, h-F449Y, and h-R477K.** Residues that are altered from the wild-type sequence are in lower case. Introduced restriction enzyme (RE) sites are underlined. Primer sequences for all other mutants used in this study are found in Tan, et al 2016[1].

**Construct**	**Primer sequence**	**Introduced RE site**
h-F241Y	GATGACCTTGAACTCTCTcGGgTaCAGCTTCGGCCATGGCCTG	*Ava*I
h-F449Y	CGGGGTGGGGGCTGCgTatACCTGCATCACCATCTACAGC	*Acc*I
h-R477K	TTGGGCCAGATGGCAGCCaagGGAGGcGCCATCCTGGGGCCTCTGG	*Kas*I

1. Tan, P. K., Ostertag, T. M. & Miner, J. N. Mechanism of high affinity inhibition of the human urate transporter URAT1. *Sci. Rep.* **6,** 34995 (2016).

Supplementary Table 2. Alignment of human URAT1 with the organic anion transporter (OAT) subfamily of the *SLC22A* family of homologs. Protein sequences were obtained from Uniprot (http://www.uniprot.org/) and aligned using the Clustal Omega multiple sequence alignment tool (http://www.ebi.ac.uk/Tools/msa/clustalo/). URAT1 residues highlighted in this study are indicated. Consensus symbols have the following meanings: An * (asterisk) indicates positions which have a single, fully conserved residue. A **:** (colon) indicates conservation between groups of strongly similar properties as below – roughly equivalent to scoring > 0.5 in the Gonnet PAM 250 matrix: STA,NEQK,NHQK,NDEQ,QHRK,MILV,MILF,HY,FYW. A **.** (period) indicates conservation between groups of weakly similar properties as below – roughly equivalent to scoring ≤ 0.5 and > 0 in the Gonnet PAM 250 matrix: CSA,ATV,SAG,STNK,STPA,SGND,SNDEQK,NDEQHK,NEQHRK,FVLIM,HFY.

PROTEIN/GENE	UNIPROTKB IDENTIFIER	SEQUENCE	RESIDUE NUMBER
		Human URAT1 Ser-35 ↓	
URAT1/SLC22A12	sp|Q96S37|S22AC_HUMAN	-MAFSELLDLVGGLGRFQVLQTMALMVSIMWLCTQSMLENFSAAVPSHRCWAPLLDNSTA	59
OAT4/SLC22A11	sp|Q9NSA0|S22AB_HUMAN	-MAFSKLLEQAGGVGLFQTLQVLTFILPCLMIPSQMLLENFSAAIPGHRCWTHMLDNGSA	59
OAT1/SLC22A6	sp|Q4U2R8|S22A6_HUMAN	-MAFNDLLQQVGGVGRFQQIQVTLVVLPLLLMASHNTLQNFTAAIPTHHCRPPADAN-LS	58
OAT3/SLC22A8	sp|Q8TCC7|S22A8_HUMAN	-MTFSEILDRVGSMGHFQFLHVAILGLPILNMANHNLLQIFTAATPVHHCRPPHNAS-TG	58
OAT5/SLC22A10	sp|Q63ZE4|S22AA_HUMAN	-MAFEELLSQVGGLGRFQMLHLVFILPSLMLLIPHILLENFAAAIPGHRCWVHMLDNNTG	59
SLC22A24	tr|C9JC66|C9JC66_HUMAN	-MGFDVLLDQVGGMGRFQICLIAFFCITNILLFPNIVLENFTAFTPSHRCWVPLLDNDTV	59
SLC22A25	sp|Q6T423|S22AP_HUMAN	-MAFQDLLDQVGGLGRFQILQMVFLIMFNVIVYHQTQLENFAAFILDHRCWVHILDNDTI	59
OAT7/SLC22A9	sp|Q8IVM8|S22A9_HUMAN	-MAFQDLLGHAGDLWRFQILQTVFLSIFAVATYLHFMLENFTAFIPGHRCWVHILDNDTV	59
SLC22A20	sp|A6NK97|S22AK_HUMAN	-MAFTDLLDALGSMGRFQLNHTALLLLPCGLLACHNFLQNFTAAVPPHHCRGPANHT-EA	58
OAT2/SLC22A7	sp|Q9Y694|S22A7_HUMAN	-MGFEELLEQVGGFGPFQLRNVALLALPRVLLPLHFLLPIFLAAVPAHRCALPGAPANFS	59
OAT10/SLC22A13	sp|Q9Y226|S22AD_HUMAN	MAQFVQVLAEIGDFGRFQIQLLILLCVLNFLSPFYFFAHVFMVLDEPHHCAVAWVKNHTF	60
consensus		* :* * . ** . * . *:*	
URAT1/SLC22A12	sp|Q96S37|S22AC_HUMAN	QASILGSLSPEALLAISIPPGPNQRPHQCRRFRQPQWQLLDPNAT-----ATSWSEADTE	114
OAT4/SLC22A11	sp|Q9NSA0|S22AB_HUMAN	V---STNMTPKALLTISIPPGPNQGPHQCRRFRQPQWQLLDPNAT-----ATSWSEADTE	111
OAT1/SLC22A6	sp|Q4U2R8|S22A6_HUMAN	KNG-----GLEV----WLPRDRQGQPESCLRFTSPQWGLPFLNGT-----EA-NGTGATE	103
OAT3/SLC22A8	sp|Q8TCC7|S22A8_HUMAN	----------PW----VLPMGPNGKPERCLRFVHPPNA-----SL-----PN-DTQRAME	93
OAT5/SLC22A10	sp|Q63ZE4|S22AA_HUMAN	SGNETGILSEDALLRISIPLDSNLRPEKCRRFVHPQWQLLHLNGT-----IHSTSEADTE	114
SLC22A24	tr|C9JC66|C9JC66_HUMAN	SDNDTGTLSKDDLLRISIPLDSNLRPQKCQRFIHPQWQLLHLNGT-----FPNTNEPDTE	114
SLC22A25	sp|Q6T423|S22AP_HUMAN	PDNDPGTLSQDALLRISIPFDSNLRPEKCRRFVHPQWKLIHLNGT-----FPNTSEPDTE	114
OAT7/SLC22A9	sp|Q8IVM8|S22A9_HUMAN	SDNDTGALSQDALLRISIPLDSNMRPEKCRRFVHPQWQLLHLNGT-----FPNTSDADME	114
SLC22A20	sp|A6NK97|S22AK_HUMAN	STN-----DSGAWLRATIPLDQLGAPEPCRRFTKPQWALLSPNS-------S-IPGAATE	105
OAT2/SLC22A7	sp|Q9Y694|S22A7_HUMAN	--------HQDVWLEAHLPREPDGTLSSCLRFAYPQALPNTTLGEERQSRGELEDEPATV	111
OAT10/SLC22A13	sp|Q9Y226|S22AD_HUMAN	------NLSAAEQLVLSVPLDTAGHPEPCLMFRPPPANASLQD-------ILSHRFNETQ	107
consensus		:* * * *	

```
URAT1/SLC22A12 sp|Q96S37|S22AC_HUMAN       PCVDGWVYDRSIFTSTIVA--KWNLVCDSHALKPMAQSIYLAGILVGAAACGPASDRFGR   172
OAT4/SLC22A11  sp|Q9NSA0|S22AB_HUMAN       PCVDGWVYDRSVFTSTIVA--KWDLVCSSQGLKPLSQSIFMSGILVGSFIWGLLSYRFGR   169
OAT1/SLC22A6   sp|Q4U2R8|S22A6_HUMAN       PCTDGWIYDNSTFPSTIVT--EWDLVCSHRALRQLAQSLYMVGVLLGAMVFGYLADRLGR   161
OAT3/SLC22A8   sp|Q8TCC7|S22A8_HUMAN       PCLDGWVYNST--KDSIVT--EWDLVCNSNKLKEMAQSIFMAGILIGGLVLGDLSDRFGR   149
OAT5/SLC22A10  sp|Q63ZE4|S22AA_HUMAN       PCVDGWVYDQSYFPSTIVT--KWDLVCDYQSLKSVVQFLLLTGMLVGGIIGGHVSDRFGR   172
SLC22A24       tr|C9JC66|C9JC66_HUMAN      PCVDGWVYDRSSFLSTIVT--EWDLVCESQSLKSMVQSLFMAGSLLGGLIYGHLSDRVGR   172
SLC22A25       sp|Q6T423|S22AP_HUMAN       PCVDGWVYDQSSFPSTIVT--KWDLVCESQPLNSVAKFLFMAGMMVGGNLYGHLSDRFGR   172
OAT7/SLC22A9   sp|Q8IVM8|S22A9_HUMAN       PCVDGWVYDRISFSSTIVT--EWDLVCDSQSLTSVAKFVFMAGMMVGGILGGHLSDRFGR   172
SLC22A20       sp|A6NK97|S22AK_HUMAN       GCKDGWVYNRSVFPSTIVM--EWDLVCEARTLRDLAQSVYMAGVLVGAAVFGSLADRLGC   163
OAT2/SLC22A7   sp|Q9Y694|S22A7_HUMAN       PCSQGWEYDHSEFSSTIATESQWDLVCEQKGLNRAASTFFFAGVLVGAVAFGYLSDRFGR   171
OAT10/SLC22A13 sp|Q9Y226|S22AD_HUMAN       PCDMGWEYPENRLPSLK---NEFNLVCDRKHLKDTTQSVFMAGLLVGTLMFGPLCDRIGR   164
consensus                                   *  ** *      .      :::***. . *    . . : * ::*    *  . *.*

URAT1/SLC22A12 sp|Q96S37|S22AC_HUMAN       RLVLTWSYLQMAVMGTAAAFAPAFPVYCLFRFLLAFAVAGVMMNTGTLLMEWTAARARPL   232
OAT4/SLC22A11  sp|Q9NSA0|S22AB_HUMAN       KPMLSWCCLQLAVAGTSTIFAPTFVIYCGLRFVAAFGMAGIFLSSLTLMVEWTTTSRRAV   229
OAT1/SLC22A6   sp|Q4U2R8|S22A6_HUMAN       RKVLILNYLQTAVSGTCAAFAPNFPIYCAFRLLSGMALAGISLNCMTLNVEWMPIHTRAC   221
OAT3/SLC22A8   sp|Q8TCC7|S22A8_HUMAN       RPILTCSYLLLAASGSGAAFSPTFPIYMVFRFLCGFGISGITLSTVILNVEWVPTRMRAI   209
OAT5/SLC22A10  sp|Q63ZE4|S22AA_HUMAN       RFILRWCLLQLAITDTCAAFAPTFPVYCVLRFLAGFSSMIIISNNSLPITEWIRPNSKAL   232
SLC22A24       tr|C9JC66|C9JC66_HUMAN      KIICKLCFLQLAISNTCAAFAPTFLVYCILRFLAGFSTMTILGNTFILSLEWTLPRSRSM   232
SLC22A25       sp|Q6T423|S22AP_HUMAN       KFVLRWSYLQLAIVGTCAAFAPTILVYCSLRFLAGAATFSIIVNTVLLIVEWITHQFCAM   232
OAT7/SLC22A9   sp|Q8IVM8|S22A9_HUMAN       RFVLRWCYLQVAIVGTCAALAPTFLIYCSLRFLSGIAAMSLITNTIMLIAEWATHRFQAM   232
SLC22A20       sp|A6NK97|S22AK_HUMAN       KGPLVWSYLQLAASGAATAYFSSFSAYCVFRFLMGMTFSGIILNSVSLVVEWMPTRGRTV   223
OAT2/SLC22A7   sp|Q9Y694|S22A7_HUMAN       RRLLLVAYVSTLVLGLASAASVSYVMFAITRTLTGSALAGFTIIVMPLELEWLDVEHRTV   231
OAT10/SLC22A13 sp|Q9Y226|S22AD_HUMAN       KATILAQLLLFTLIGLATAFVPSFELYMALRFAVATAVAGLSFSNVTLLTEWVGPSWRTQ   224
consensus                                  :       :        :        :   *   .     .         **

                                           Human URAT1 F241
                                                   ↓
URAT1/SLC22A12 sp|Q96S37|S22AC_HUMAN       VMTLNSLGFSFGHGLTAAVAYGVRDWTLLQLVVSVPFFLCFLYSWWLAESARWLLTTGRL   292
OAT4/SLC22A11  sp|Q9NSA0|S22AB_HUMAN       TMTVVGCAFSAGQAALGGLAFALRDWRTLQLAASVPFFAISLISWWLPESARWLIIKGKP   289
OAT1/SLC22A6   sp|Q4U2R8|S22A6_HUMAN       VGTLIGYVYSLGQFLLAGVAYAVPHWRHLQLLVSAPFFAFFIYSWFFIESARWHSSSGRL   281
OAT3/SLC22A8   sp|Q8TCC7|S22A8_HUMAN       MSTALGYCYTFGQFILPGLAYAIPQWRWLQLTVSIPFFVFFLSSWWTPESIRWLVLSGKS   269
OAT5/SLC22A10  sp|Q63ZE4|S22AA_HUMAN       VVILSSGALSIGQIILGGLAYVFRDWQTLHVVASVPFFVFFLLSRWLVESARWLIITNKL   292
SLC22A24       tr|C9JC66|C9JC66_HUMAN      TIMVLLCSYSVGQMLLGGLAFAIQDWHILQLTVSTPIIVLFLSSWKMVESARWLIINNQL   292
SLC22A25       sp|Q6T423|S22AP_HUMAN       ALTLTLCAASIGHITLGSLAFVIRDQCILQLVMSAPCFVFFLFSRWLAESARWLIINNKP   292
OAT7/SLC22A9   sp|Q8IVM8|S22A9_HUMAN       GITLGMCPSGIAFMTLAGLAFAIRDWHILQLVVSVPYFVIFLTSSWLLESARWLIINNKP   292
SLC22A20       sp|A6NK97|S22AK_HUMAN       AGILLGYSFTLGQLILAGVAYLIRPWRCLQFAISAPFLIFFLYSWWLPESSRWLLLHGKS   283
OAT2/SLC22A7   sp|Q9Y694|S22A7_HUMAN       AGVLSSTFWTGGVMLLALVGYLIRDWRWLLLAVTLPCAPGILSLWWVPESARWLLTQGHV   291
OAT10/SLC22A13 sp|Q9Y226|S22AD_HUMAN       AVVLAQCNFSLGQMVLAGLAYGFRNWRLLQITGTAPGLLLFFYFWALPESARWLLTRGRM   284
consensus                                             .      :.: .     * .  : *     :      ** **     :
```

```
URAT1/SLC22A12 sp|Q96S37|S22AC_HUMAN     DWGLQELWRVAAINGKGAVQDTLTPEVLLSAMREELSMGQP-PASLGTLLRMPGLRFRTC  351
OAT4/SLC22A11  sp|Q9NSA0|S22AB_HUMAN     DQALQELRKVARINGHKEAK-NLTIEVLMSSVKEEVASAKE-PRSVLDLFCVPVLRWRSC  347
OAT1/SLC22A6   sp|Q4U2R8|S22A6_HUMAN     DLTLRALQRVARINGKREEGAKLSMEVLRASLQKELTMGKG-QASAMELLRCPTLRHLFL  340
OAT3/SLC22A8   sp|Q8TCC7|S22A8_HUMAN     SKALKILRRVAVFNGKKEEGERLSLEELKLNLQKEISLAKA-KYTASDLFRIPMLRRMTF  328
OAT5/SLC22A10  sp|Q63ZE4|S22AA_HUMAN     DEGLKALRKVARTNGIKNAEETLNIEVVRSTMQEELDAAQT-KTTVCDLFRNPSMRKRIC  351
SLC22A24       tr|C9JC66|C9JC66_HUMAN    DEGLKELRRVAHINGKKNTEETLTTELVRSTMKKELDAVRI-KTSIFSLFRAPKLRMRVF  351
SLC22A25       sp|Q6T423|S22AP_HUMAN     EEGLKELRKAAHRNGMKNAEDILTMEVLKSTMKQELEAAQK-KHSLCELLRIPNICKRIC  351
OAT7/SLC22A9   sp|Q8IVM8|S22A9_HUMAN     EEGLKELRKAAHRSGMKNARDTLTLEILKSTMKKELEAAQKKKPSLCEMLHMPNICKRIS  352
SLC22A20       sp|A6NK97|S22AK_HUMAN     QLAVQNLQKVAAMNGRKEEGERLTKEVMSSYIQSEFASVCT-SNSILDLFRTPAIRKVTC  342
OAT2/SLC22A7   sp|Q9Y694|S22A7_HUMAN     KEAHRYLLHCARLNGRPVCEDSFSQEAVSKVAAGERVV---RRPSYLDLFRTPRLRHISL  348
OAT10/SLC22A13 sp|Q9Y226|S22AD_HUMAN     DEAIQLIQKAASVNRRKLSPELMNQ-----LVPEKT----GPSGNALDLFRHPQLRKVTL  335
consensus                                .   : : : *  .        :.          :         .   ::  * :

                                              Human URAT1 F365
                                                      ↓
URAT1/SLC22A12 sp|Q96S37|S22AC_HUMAN     ISTLCWFAFGFTFFGLALDLQALGSNIFLLQMFIGVVDIPAKMGALLLLSHLGRRPTLAA  411
OAT4/SLC22A11  sp|Q9NSA0|S22AB_HUMAN     AMLVVNFSLLISYYGLVFDLQSLGRDIFLLQALFGAVDFLGRATTALLLSFLGRRTIQAG  407
OAT1/SLC22A6   sp|Q4U2R8|S22A6_HUMAN     CLSMLWFATSFAYYGLVMDLQGFGVSIYLIQVIFGAVDLPAKLVGFLVINSLGRRPAQMA  400
OAT3/SLC22A8   sp|Q8TCC7|S22A8_HUMAN     CLSLAWFATGFAYYSLAMGVEEFGVNLYILQIIFGGVDVPAKFITILSLSYLGRHTTQAA  388
OAT5/SLC22A10  sp|Q63ZE4|S22AA_HUMAN     ILVFLRFANTIPFYGTMVNLQHVGSNIFLLQVLYGAVALIVRCLALLTLNHMGRRISQIL  411
SLC22A24       tr|C9JC66|C9JC66_HUMAN    GLCFVRFAITVPFYGLILNLQHLGSNVSLFQILCGAVTFTARCVSLLTLNHMGRRISQIL  411
SLC22A25       sp|Q6T423|S22AP_HUMAN     FLSFVRFASTIPFWGLTLHLQHLGNNVFLLQTLFGAVTLLANCVAPWALNHMSRRLSQML  411
OAT7/SLC22A9   sp|Q8IVM8|S22A9_HUMAN     LLSFTRFANFMAYFGLNLHVQHLGNNVFLLQTLFGAVILLANCVAPWALKYMNRRASQML  412
SLC22A20       sp|A6NK97|S22AK_HUMAN     CLMVIWFSNSVAYYGLAMDLQKFGLSLYLVQALFGIINTPAMLVATATMIYVGRRATVAS  402
OAT2/SLC22A7   sp|Q9Y694|S22A7_HUMAN     CCVVVWFGVNFSYYGLSLDVSGLGLNVYQTQLLFGAVELPSKLLVYLSVRYAGRRLTQAG  408
OAT10/SLC22A13 sp|Q9Y226|S22AD_HUMAN     IIFCVWFVDSLGYYGLSLQVGDFGLDVYLTQLIFGAVEVPARCSSIFMMQRFGRKWSQLG  395
consensus                                      *   . ::.  . :  .* .:   * : * :           :    *:

                                                                       Human URAT1 F449
                                                                             ↓
URAT1/SLC22A12 sp|Q96S37|S22AC_HUMAN     SLLLAGLCILANTLVPHEMGALRSALAVLGLGGVGAAFTCITIYSSELFPTVLRMTAVGL  471
OAT4/SLC22A11  sp|Q9NSA0|S22AB_HUMAN     SQAMAGLAILANMLVPQDLQTLRVVFAVLGKGCFGISLTCLTIYKAELFPTPVRMTADGI  467
OAT1/SLC22A6   sp|Q4U2R8|S22A6_HUMAN     ALLLAGICILLNGVIPQDQSIVRTSLAVLGKGCLAASFNCIFLYTGELYPTMIRQTGMGM  460
OAT3/SLC22A8   sp|Q8TCC7|S22A8_HUMAN     ALLLAGGAILALTFVPLDLQTVRTVLAVFGKGCLSSSFSCLFLYTSELYPTVIRQTGMGV  448
OAT5/SLC22A10  sp|Q63ZE4|S22AA_HUMAN     FMFLVGLSILANTFVPKEMQTLRVALACLGIGCSAATFSSVAVHFIELIPTVLRARASGI  471
SLC22A24       tr|C9JC66|C9JC66_HUMAN    FTFPVGLFILVNTFLPQEMQILRVVLATLGIGSVSAASNSASVHHNELVPTILRSTVAGI  471
SLC22A25       sp|Q6T423|S22AP_HUMAN     LMFLLATCLLAIIFVPQEMQTLRVVLATLGVGAASLGITCSTAQENELIPSIIRGRATGI  471
OAT7/SLC22A9   sp|Q8IVM8|S22A9_HUMAN     LMFLLAICLLAIIFVPQEMQTLREVLATLGLGASALANTLAFAHGNEVIPTIIRARAMGI  472
SLC22A20       sp|A6NK97|S22AK_HUMAN     FLILAGLMVIANMFVPEGTQILCTAQAALGKGCLASSFICVYLFTGELYPTEIRQMGMGF  462
OAT2/SLC22A7   sp|Q9Y694|S22A7_HUMAN     TLLGTALAFGTRLLVSSDMKSWSTVLAVMGKAFSEAAFTTAYLFTSELYPTVLRQTGMGL  468

OAT10/SLC22A13 sp|Q9Y226|S22AD_HUMAN     TLVLGGLMCIIIIFIPADLPVVVTMLAVVGKMATAAAFTISYVYSAELFPTILRQTGMGL  455
consensus                                     .       .:           * .*                *: *: :*    *

                               Human URAT1 R477   Human URAT1 I481
                                              ↓   ↓
URAT1/SLC22A12 sp|Q96S37|S22AC_HUMAN     GQMAARGGAILGPLVRLLGVHGPWLPLLVYGTVPVLSGLAA-LLLPETQSLPLPDTIQDV  530
OAT4/SLC22A11  sp|Q9NSA0|S22AB_HUMAN     LHTVGRLGAMMGPLILMSRQALPLLPPLLYGVISIASSLVVLFFLPETQGLPLPDTIQDL  527
OAT1/SLC22A6   sp|Q4U2R8|S22A6_HUMAN     GSTMARVGSIVSPLVSMTAELYPSMPLFIYGAVPVAASAVT-VLLPETLGQPLPDTVQDL  519
OAT3/SLC22A8   sp|Q8TCC7|S22A8_HUMAN     SNLWTRVGSMVSPLVKITGEVQPFIPNIIYGITALLGGSAA-LFLPETLNQPLPETIEDL  507
OAT5/SLC22A10  sp|Q63ZE4|S22AA_HUMAN     DLTASRIGAALAPLLMTLTVFFTTLPWIIYGIFPIIGGLIV-FLLPETKNLPLPDTIKDV  530
SLC22A24       tr|C9JC66|C9JC66_HUMAN    NAVSGRTGAALAPLLMTLMAYSPHLPWISYGVFPILAVPVI-LLLPETRDLPLPNTIQDV  530
SLC22A25       sp|Q6T423|S22AP_HUMAN     TGNFANIGGALASLMMILSIYSRPLPWIIYGVFAILSGLVV-LLLPETRNQPLLDSIQDV  530
OAT7/SLC22A9   sp|Q8IVM8|S22A9_HUMAN     NATFANIAGALAPLMMILSVYSPPLPWIIYGVFPFISGFAF-LLLPETRNKPLFDTIQDE  531
SLC22A20       sp|A6NK97|S22AK_HUMAN     ASVHARLGGLTAPLVTTLGEYSTILPPVSFGATAILAGLAV-CVLTETRNMPLVETIAAM  521
OAT2/SLC22A7   sp|Q9Y694|S22A7_HUMAN     TALVGRLGGSLAPLAALLDGVWLSLPKLTYGGIALLAAGTA-LLLPETRQAQLPETIQDV  527
OAT10/SLC22A13 sp|Q9Y226|S22AD_HUMAN     VGIFSRIGGILTPLVILLGEYHAALPMLIYGSLPIVAGLLC-TLLPETHGQGLKDTLQDL  514
consensus                                     . ..    *          :* . :*   . .      .* **    * :::

URAT1/SLC22A12 sp|Q96S37|S22AC_HUMAN     QNQAVKKATHGTLGNSVLKSTQF---------------------------            553
OAT4/SLC22A11  sp|Q9NSA0|S22AB_HUMAN     ESQKSTAAQGNRQEAVTVESTSL---------------------------            550
OAT1/SLC22A6   sp|Q4U2R8|S22A6_HUMAN     ESRWAPTQKEAGIYPRKGKQTRQQQEHQKYMVPLQASAQEKNGL-----            563
OAT3/SLC22A8   sp|Q8TCC7|S22A8_HUMAN     EN-WSLRAKKPKQEPEVEK--------ASQRIPLQPHGPGLGSS-----            542
OAT5/SLC22A10  sp|Q63ZE4|S22AA_HUMAN     ENQKKN-L-KEKA------------------------------------            541
SLC22A24       tr|C9JC66|C9JC66_HUMAN    ENEKDS-R-NIKQEDTCMKVTQF---------------------------            551
SLC22A25       sp|Q6T423|S22AP_HUMAN     ENEGVNSL-AAPQRSSVL-------------------------------            547
OAT7/SLC22A9   sp|Q8IVM8|S22A9_HUMAN     KNERKDPR-EPKQEDPRVEVTQF---------------------------            553
SLC22A20       sp|A6NK97|S22AK_HUMAN     ERRVKEGSSKKHVEEKSEEISLQQ----LRASPLKETI-----------            555
OAT2/SLC22A7   sp|Q9Y694|S22A7_HUMAN     ERKSAPTSLQEE--EMPMKQ--VQN------------------------            548
OAT10/SLC22A13 sp|Q9Y226|S22AD_HUMAN     ELGPHPRSPKSVPSE---KETEAKGRT---------SSPGVAFVSSTYF            551
consensus                                :
```
